# Sammba-MRI: A Library for Processing SmAll-MaMmal BrAin MRI Data in Python

**DOI:** 10.3389/fninf.2020.00024

**Published:** 2020-05-28

**Authors:** Marina Celestine, Nachiket A. Nadkarni, Clément M. Garin, Salma Bougacha, Marc Dhenain

**Affiliations:** ^1^UMR9199 Laboratory of Neurodegenerative Diseases, Centre National de la Recherche Scientifique (CNRS), Fontenay-aux-Roses, France; ^2^MIRCen, Institut de Biologie François Jacob, Commissariat à l'Energie Atomique et aux Energies Alternatives (CEA), Fontenay-aux-Roses, France; ^3^UMR-S U1237 Physiopathologie et imagerie des troubles Neurologiques (PhIND), INSERM, Université de Caen-Normandie, GIP Cyceron, Caen, France; ^4^Normandie Université, UNICAEN, PSL Research University, EPHE, Inserm, U1077, CHU de Caen, Neuropsychologie et Imagerie de la Mémoire Humaine, Caen, France

**Keywords:** processing pipeline, MRI, registration, small animal neuroimaging, Python

## Abstract

Small-mammal neuroimaging offers incredible opportunities to investigate structural and functional aspects of the brain. Many tools have been developed in the last decade to analyse small animal data, but current softwares are less mature than the available tools that process human brain data. The Python package Sammba-MRI (SmAll-MaMmal BrAin MRI in Python; http://sammba-mri.github.io) allows flexible and efficient use of existing methods and enables fluent scriptable analysis workflows, from raw data conversion to multimodal processing.

## 1. Introduction

The use of magnetic resonance imaging (MRI) methods in animals provides considerable benefits for improving our understanding of brain structure and function in health and diseases. The greatest advantages of preclinical MRI include group homogeneity and the opportunity to acquire a high amount of information repeated as needed. This added value, together with practical and ethical considerations, resulted in an increase of the use of small-mammal MRI in research. In human brain imaging, a large variety of high level software solutions is available for MRI preprocessing and analysis (e.g., SPM^1^, FSL^2^, or AFNI^3^). Less Free and Open Source Software (FOSS) are already available to analyse animal MRI. Atlas-based Imaging Data Analysis of structural and functional mouse brain MRI (AIDAmri) (Pallast et al., 2019) package allows registration of functional and diffusion mouse brain MRI with the Allen Mouse Brain Atlas (Allen Institute for Brain Science, 2004; Lein et al., 2007). The SAMRI (Small Animal Magnetic Resonance Imaging) package provides fMRI preprocessing, metadata parsing, and data analysis functions optimized for mouse brains (Ioanas et al., 2020). Today, there is still a need for other efficient and collaborative tools that would facilitate the adoption and dissemination of standardized pre-processing strategies for small animal MRI. Sammba-MRI was designed to process MR images, including anatomical, functional, and perfusion images. It allows to preprocess image dataset (conversion to NIfTI, bias correction), register images to templates or atlases, and perform perfusion measures.

## 2. Workflow

### 2.1. Tools: Python Ecosystem and Neuroimaging Software Packages

With its FOSS dependency stack and its growing neuroimaging community Python has been naturally the language of choice for our package. The scientific Python libraries used in Sammba-MRI are NumPy (Oliphant, 2006), SciPy (Millman and Aivazis, 2011), the neuroimaging data analysis tools nibabel^4^, Nilearn (Abraham et al., 2014) and Nipype (Gorgolewski et al., 2011). Visualization functionality depends on Matplotlib (Hunter, 2007) or Graphviz (Gansner and North, 2000), but neither is required to perform MRI data processing.

Via Nipype, we utilize basic MRI preprocessing functions from AFNI (Cox, 1996), FSL (Jenkinson et al., 2012) and ANTs (Avants et al., 2009) packages. The dependency on the efficient but non open-source brain segmentation RATs tool (Oguz et al., 2014) is optional.

More specifically, Sammba-MRI and the examples provided in its manual depends on the following libraries: Nipype ≥ 1.0.4; Nilearn ≥ 0.4.0; Numpy ≥ 1.14; SciPy ≥ 0.19; Nibabel ≥ 2.0.2; Sklearn ≥ 0.19; matplotlib ≥ 1.5.1; nose ≥ 1.2.1; doctest-ignore-unicode; DICOM ToolKit package as well as FSL (version 5.0), AFNI, ANTs, and RATS.

### 2.2. Code Design

Sammba-MRI is developed within GitHub development platform^5^. Coding guidelines follow the model of Nilearn and other successfully adopted packages (e.g., Scikit-learn Pedregosa et al., 2011) to make the codebase understandable and easily maintainable^6^. Objects are used with parsimony: the different registration classes share all the same interface, and the brain extraction classes comply to the Nipype BaseInterface.

Effort is made to keep the code uniformly formatted and to use consistent naming for the functions and parameters following the coding conventions of Nilearn. Preprocessing building blocks and pipelines are automatically tested on light MRI data samples to ensure code quality. Finally, the user is guided through Sammba-MRI with extensive documentation including installation instructions, application programming interface (API) reference, pipeline graphs, and practical examples based on publicly available small animal neuroimaging datasets.

**Figure 1:**
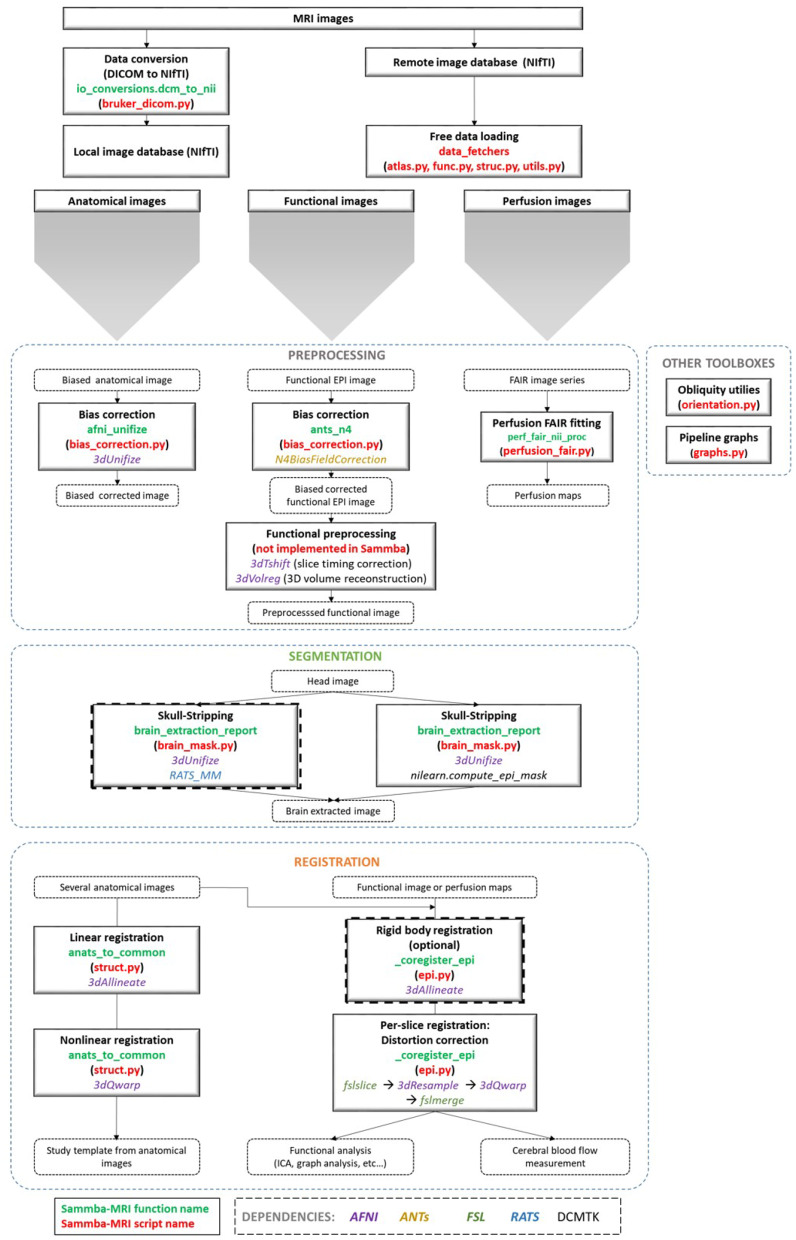
Sammba-MRI workflow. Anatomical, functional, or perfusion images are imported from MRI local scanners or databases. They are analyzed using different preprocessing, segmentation and registration modules. Each function (green) and python-scripts (red) of Sammba-MRI are presented. Library dependencies are specified with color codes.

An overview of the modules used to manipulate images is presented in Figure 1. These modules are implemented either as “stand-alone” (i.e., bias_correction) or as ready-to-use pipelines (i.e., TemplateRegistrator).

### 2.3. DICOM to NIfTI Conversion

Sammba-MRI allows to convert Bruker DICOM (digital imaging and communications in medicine) files to the standard Neuroimaging Informatics Technology Initiative format (NIfTI-1) and extracts extensive information using DCMTK package (Eichelberg et al., 2004). Bruker files conversion is an active development field, with various available tools handling DICOM (e.g., dicomifier^7^) or not (e.g., bru2nii^8^, Bruker2nifti^9^, bruker2nifti^10^). Finally, ParaVision 360 with the latest patch 1.1 can export the NIFTI format since February 2019. Our implementation is meant to be a light helper function, allowing to handle the conversion on the fly. It has been tested only for Paravision 6 and a limited number of imaging sequences.

### 2.4. Bias Field Correction

Intensity non-uniformity modeling is essential in preclinical studies because the intensity gradient corrupting MR images becomes particularly pronounced at high field strengths (Boyes et al., 2008). Sammba-MRI relies on AFNI's 3dUnifize to correct for intensity bias in anatomical images, and on N4BiasFieldCorrection function of the ANTs package (Tustison et al., 2010) for the other modalities. 3dUnifize is also used to aid brain extraction, as detailed in the following paragraph.

### 2.5. Skull-Stripping

Skull-stripping is a critical early step in processing MR images from small animals. Various automatic rodent-specific softwares (Chou et al., 2011; Oguz et al., 2014) or adaptations of human algorithms (Wood et al., 2013; AFNI's 3dskullstrip -rat) are freely available for research purposes. We choose to rely on the LOGISMOS-based graph segmentation (Yin et al., 2010) based on grayscale mathematical morphology RATS software (Oguz et al., 2014) because of its good performance across a wide range of datasets (Sargolzaei et al., 2018). An alternative to the free but non-open source RATS tool is also available, based on an adaptation of the human histogram-based brain extraction method of Nilearn. This method can be used in any pipeline by setting the parameter use_rats_tool to False. Because intensity inhomogeneity can hamper the performance of automatic skull-stripping, prior bias field correction is usually recommended (Sled et al., 1998) and is performed by default with 3dUnifize. The helper function brain_segmentation_report from Sammba-MRI segmentation module allows to efficiently tune the initial intensity threshold used in bias correction by producing for a given set of thresholds 5 informative measures characterizing the extracted mask to bypass time consuming repetitive visual checks. The returned features consist of the total volume of the extracted mask, its anteroposterior length, its right-left width, and its inferior-superior height as well as the sample Pearson's product-moment correlation coefficient between the brain mask image and its reflection with respect to the estimated mid-sagittal plane (Powell et al., 2016).

### 2.6. Registrations

Several registration algorithms are implemented within Sammba-MRI. First, rigid-body registration can be performed to roughly align individual images from the same modality or from different modalities. It minimizes normalized mutual information between brain extracted images. This registration is finally estimated and applied to the whole head images. Second, linear registration estimates linear transforms between a source image and a reference image. It relies on AFNI's 3dAllineate function. Linear registration is more efficient when performed on brain-extracted rather than on whole head images. Third, non-linear registration (piecewise polynomial *C*^1^ diffeomorphism) between a source image and a reference image can also performed. It relies on AFNI's 3dQwarp and iterations toward patch size reduction until a maximum refinement “level” is reached. Unlike linear registration, it is more efficient when computed using whole head images.

#### 2.6.1. Group-Wise Registration and Study-Template

Group-wise registration aims to align all images from different animals within a common space, resulting in an average brain (study template) that represents the commonalities among individual brain anatomies of a particular population. Using a study template eliminates possible bias toward external features and improves subsequent analyses (De Feo and Giove, 2019). Sammba-MRI implements the multi-level, iterative scheme proposed by Kovačević et al. (2005) to create a fine anatomical template from individual anatomical MRI scans. A first rough template is obtained by averaging bias corrected head images centered on their respective brain mask centroids. Then the individual images are registered to this template. This process of successive averaging/registration is iterated while increasing the number of degrees of freedom of the estimated transform and updating the target template (see Nadkarni et al., 2019 for a detailed description of the pipeline).

#### 2.6.2. Inter-Modality Registration

Several multimodal images can be recorded from a single animal, including structural imaging with different contrasts, blood-oxygenation-level-dependent (BOLD) and arterial spin labeling (ASL) MRI. BOLD imaging is largely used to investigate brain function in response to specific tasks or in the absence of explicit tasks (i.e., in resting state conditions) (Glover, 2011). ASL is an attractive method to image the vascular system by directly measuring blood flow (Kober et al., 2008).

In addition to the inherent difficulties in intermodality registration (Ashburner and Friston, 1997), severe image artifacts can corrupt BOLD or ASL scans resulting in a low signal-to-noise ratio (SNR). For instance, the echo planar imaging (EPI) technique widely used in functional and perfusion imaging suffers from non-linear geometric and intensity distortions caused by static magnetic field inhomogeneity that worsen at higher field strengths (Hong et al., 2015).

Thus a specific module called _coregister_epi was developed to register anatomical and EPI scans from individual animals. Anatomical images are first reoriented to match EPI images. Next, the reoriented anatomical images and the EPI scans are split into 2D slices along the z-direction (according to the slice geometry of EPI). Each EPI slice then undergoes a non-linear registration to match the corresponding anatomical slice. This per-slice registration corrects for EPI distortion while being more conservative than a global 3D non-linear registration.

## 3. Pipelines

Sammba-MRI proposes two ready-to-use pipelines to perform spatial registrations to a population or standard reference template as well as inter-modalities registration between anatomical, functional, or perfusion images. These pipelines have been tested throughout the different stages of their development process on various datasets from mice, rats and mouse lemurs and used in several publications from our lab (Garin et al., 2018, 2019; Nadkarni et al., 2019). The two pipelines are called Coregistrator and TemplateRegistrator.

All pipelines start with bias field correction for the individual images, involve skull-stripping and specific registration algorithms depending on image modality.

### 3.1. Registration Between Anatomical Images and Another Modality:Coregistrator

**Figure 2:**
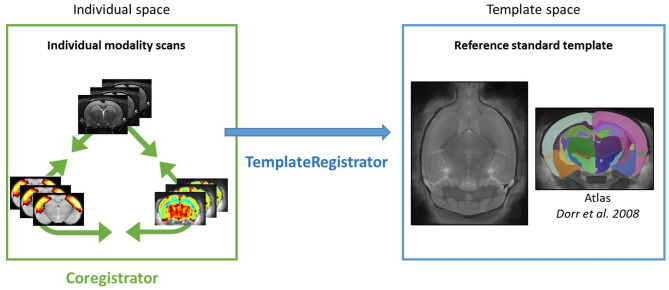
Sammba-MRI pipelines. Color box represents spaces in which individual images are registered. Registration between individual modalities is performed by Coregistrator class (green arrow). Registration of individual modality images to a reference standard template space is performed by TemplateRegistrator class (blue arrow).

Intra-subject registration between an anatomical scan and another modality (BOLD or ASL) is handled in the individual space through the Coregistrator class from the registration module (Figure 2).


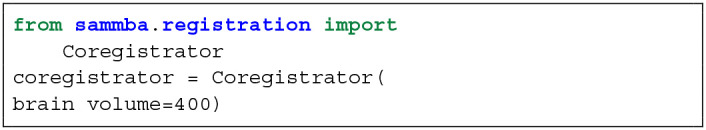


Multimodal processing slightly differs between modality. Thus, user can choose modality of interest and the critical parameters that lead to the best registration.

BOLD scans are preprocessed using the same usual steps for human data with optional slice timing correction, bias field correction, realignment to the first volume and computation of the temporal mean of all the volumes. The corresponding structural scan is then registered to the average BOLD scan. Since this is a critical step, the user can choose either to pursue with human-like pipeline by estimating a rigid body functional-to-structural transform and applying its inverse to the structural image, or to assume that the head motion between the two scans is negligible. In all cases, it is better to only reorient the anatomical image to match the modality of interest. Finally, per-slice-based registration is performed as described in *section 2.6.2*.


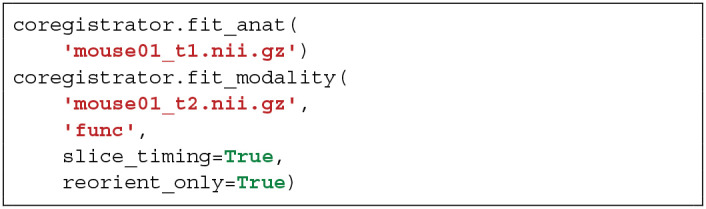


Sammba-MRI was also designed to analyse ASL scans to perform perfusion measures. This analysis relies on Bruker-FAIR (Flow-sensitive Alternating Inversion Recovery) EPI sequences. Quantitative CBF maps are first estimated using perf_fair_nii_proc function from the modality_processor module. Then Sammba-MRI allows to preprocess functional ASL scans with the equilibrium magnetization maps (M0) used as the representative volume for registration. The M0 volume is aligned to the anatomical, first with a rigid body registration and then on a per-slice basis.


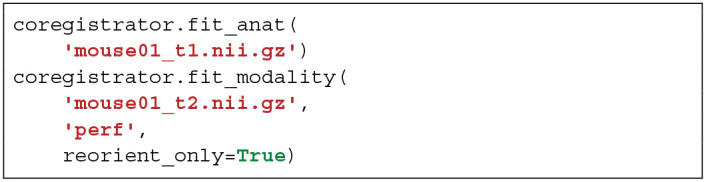


### 3.2. Template-Based Multi-Modal Processing: TemplateRegistrator

Multimodal images (anatomical, functional, or perfusion MRI) can be handled in the template space through the TemplateRegistrator class. This pipeline matches individual images to a reference template, a necessary step for group studies (Figure 2).

#### 3.2.1. Template Matching

Sammba-MRI proposes to download reference templates both for mouse and rat brains. The user needs to specify the path to the template of his choice to the TemplateRegistrator class from the registration module.


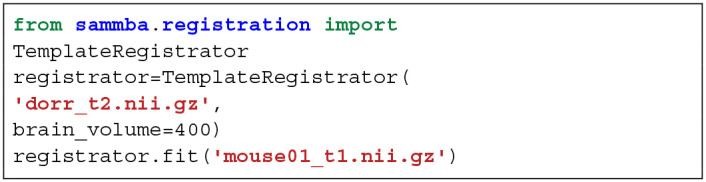


#### 3.2.2. BOLD and ASL Preprocessing

BOLD and ASL preprocessing can also be performed in template space with TemplateRegistrator. The structural-to-template warp, the functional-to-structural rigid body transform and the perslice functional-to-structural warps are combined and applied in a one-big-step transformation to the functional data to minimize interpolation errors. The TemplateRegistrator class encompasses an inverse_transform_towards_modality method to bring an image from the reference space to the individual's space.

## 4. Results

Sammba-MRI is available through the GitHub platform^11^ and was tested using different image datasets.

### 4.1. Group-Wise Registration, Registration of Anatomical Images to a Common Space, and Template Creation

**Figure 3:**
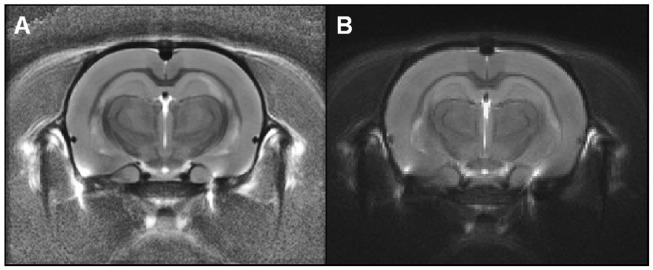
Rat templates issued from Sammba-MRI **(A)** or SPM-based **(B)** registrations. These templates were calculated from anatomical images of 10 animals. Visual observation suggests similar quality of the two templates.

First, we evaluated group-wise registration and template creation using a dataset of *in vivo* T2-weighted images of 10 Sprague-Dawley rat brains (Lancelot et al., 2014). The scans were acquired using a 7.0 T Bruker scanner at 100 × 100 × 500 μm resolution using 30 different slices. We used anats_to_common to register images from the different animals and create a group average template (Figure 3).

For comparison purposes, the registration between images from each animal was also performed using algorithms from SPM8^12^ with the SPMMouse toolbox^13^ (Sawiak et al., 2009), a reference method for image-registrations. The brain images were segmented into gray (GM) and white matter (WM) tissue probability maps using locally developed priors, then spatially transformed to a standard space. Priors were based on 100 × 100 × 100 μm resolution images and 134 slices. Affine regularization was set for an average-sized template, with a bias non-uniformity FWHM cut off of 10mm, a 5mm basis-function cut off and sampling distance of 0.3 mm. The resulting GM and WM portions were output in rigid template space, and DARTEL (Ashburner, 2007) was used to create non-linearly registered maps for each animal and common templates for the cohort of animals. The deformation fields were applied to the MR images of each animal and the resulting images were averaged to create a template. Figure 3 shows the template obtained with SPM/Dartel. No obvious difference could be identified between the two templates.

**Figure 4:**
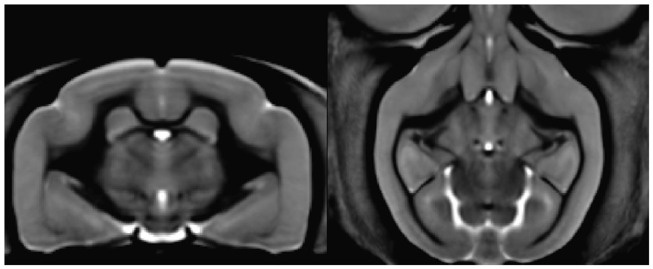
Mouse lemur template from 34 animals. Coronal section of the mouse lemur MRI template (level of hippocampus).

Sammba-MRI adapts to different small animal species. Figure 4 shows a template of mouse lemurs as another example (Nadkarni et al., 2018).

### 4.2. Validation of Template Matching

The Sprague-Dawley dataset is associated to brain segmentations into 28 regions for each animal (Lancelot et al., 2014). It also includes a study-template and an atlas based on segmentation of this template into 28 regions. Each image of the 12 individual animal was registered to the template using Sammba-MRI and the deformation fields were applied to the segmented images of each animal. We then measured the regional overlap between each region of the transformed atlases of each animal and the template-segmentation using Dice similarity coefficient ($2\frac{\left|A\cap B\right|}{\left|A|+|B\right|}$).

The deformation fields calculated with SPM were also applied to the MR and segmented images of each animal. We also measured the regional overlap between each region of the SPM-transformed segmentations of each animal and the template-segmentation also using Dice similarity coefficient.

**Figure 5:**
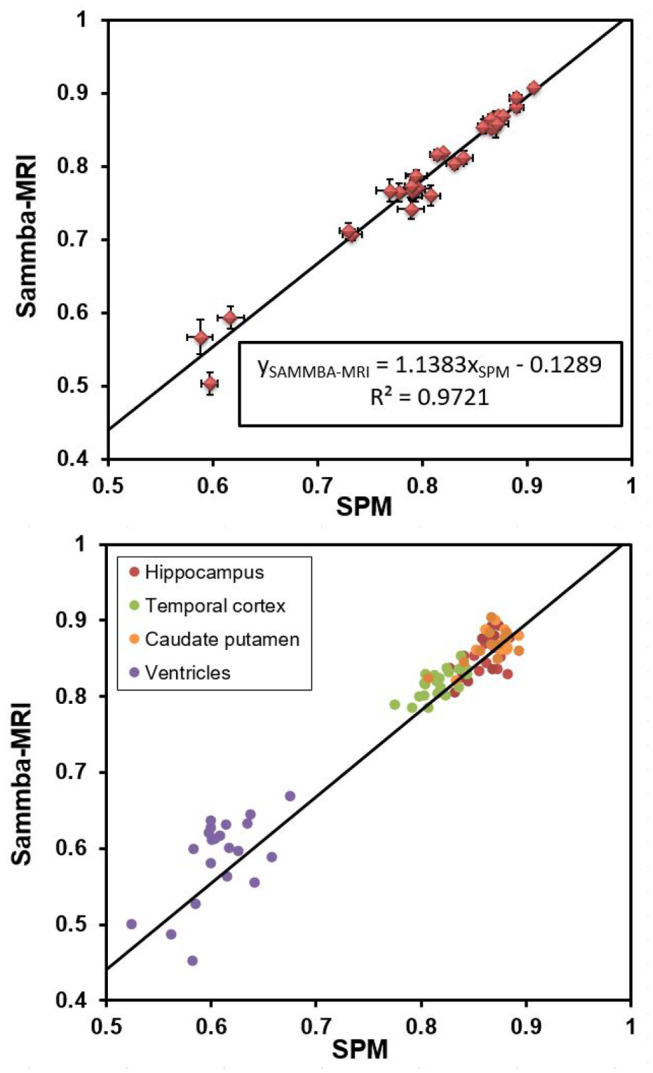
Dice coefficients obtained after registering individual images to a rat brain templates with Sammba-MRI and SPM/Dartel. **(Top)** Comparisons showing 27 brains regions. Bars represents standard error of the mean. **(Bottom)** Individual measures for four different brain regions.

Figure 5 shows that Dice coefficients obtained with Sammba-MRI were highly correlated with those obtained using SPM mouse and outperformed those of SPM in several cases (points above the line). Regions with lower Dice values correspond to ventricles.

### 4.3. fMRI and Perfusion Modalities

**Figure 6:**
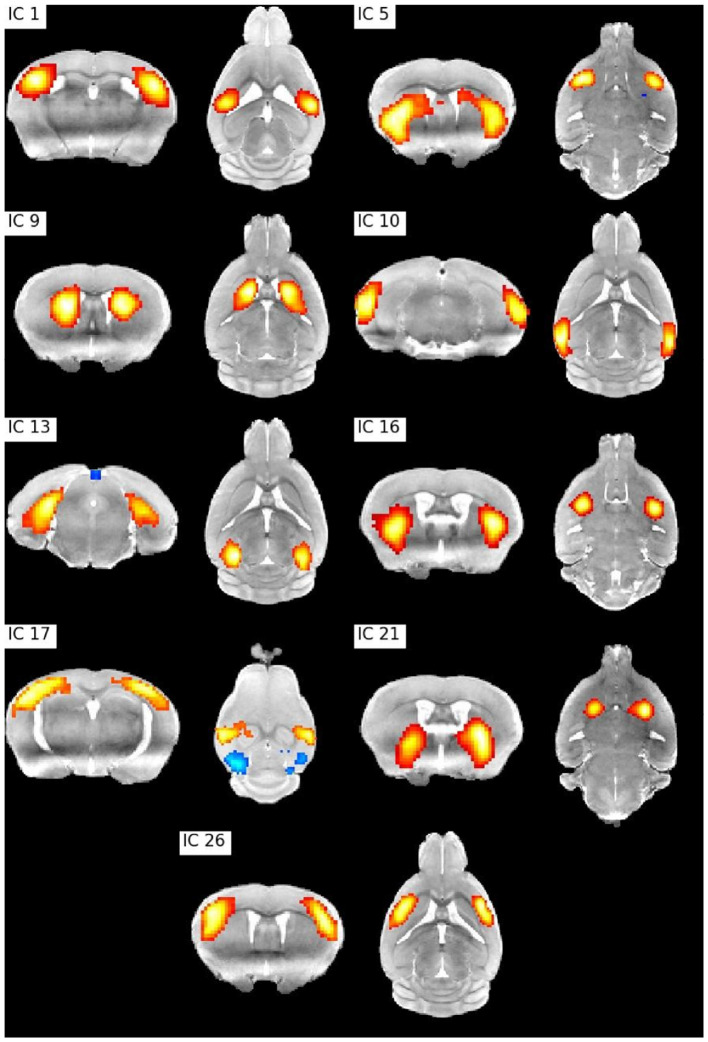
ICA bilateral components. IC 1: Barrel field (i) cortex, IC 5: Lateral striatum, IC 9: Dorsal striatum (i), IC 10: Visual cortex, IC1 3: Hippocampus, IC 16: Dorsal striatum (ii), IC 17: Barrel field (ii) cortex, IC 21: Ventral striatum, IC 26: Supplementary cortex.

Resting state fMRI allows to study temporally synchronized BOLD oscillations reflecting functionally connected brain networks. As in human resting state fMRI, spatial networks can be extracted using Independent Components Analysis (ICA) (Zerbi et al., 2015; Grandjean et al., 2020). We preprocessed the publicly shared functional data from 15 mice (2–3 months old) from (Zerbi et al., 2015) paper with Sammba-MRI and performed a group ICA (Varoquaux et al., 2010) with 30 components. Relevant bilateral regions related to somatosensory, hippocampal, visual, basal ganglia, and sensorimotor networks were obtained without additional data post-processing (Figure 6).

**Figure 7:**
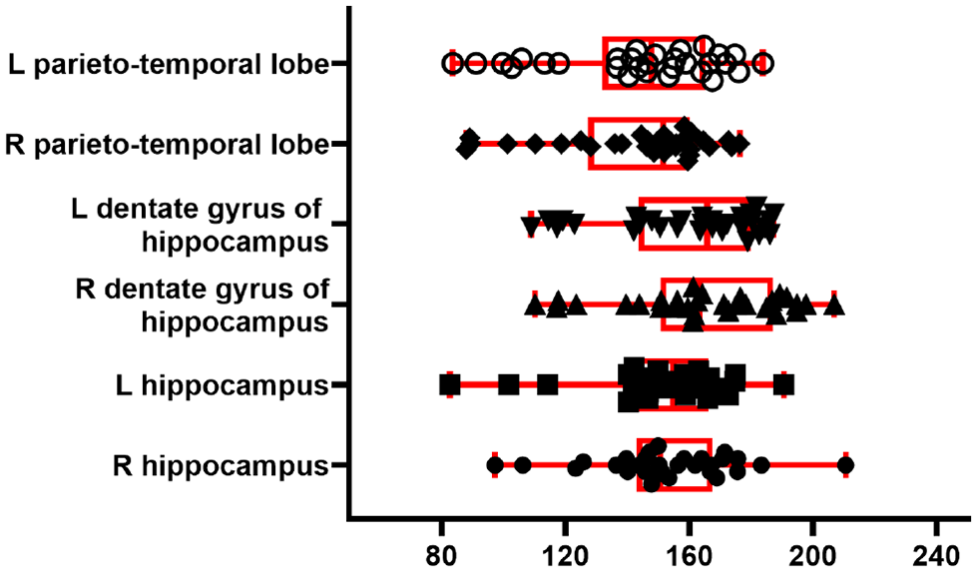
CBF from 30 C57BL/6J mice. Boxplot shows median, interquartile range, upper and lower adjacent values for six brain region. Each dot represents regional CBF in ml/100g/min from one animal.

To illustrate the perfusion processing pipeline, we used perfusion FAIR images from 30 C57BL/6J mice (6–8 months) to quantify CBF. Figure 7 shows regional absolute CBF values. Perfusion values of 152±22 and 143±26 ml/100g/min in the hippocampus and temporal cortex, respectively. These values are lower than those reported by Kober et al. (208±20 and 243±35 ml/100g/min in the hippocampus and cortex) with FAIR method (Kober et al., 2008). They are higher than those (118±6 ml/100g/min in the cortex) reported with the same method by (Zheng et al., 2010).

## 5. Big Data, Reproducibility, Collaboration

The package design facilitates big data exploration: the user is able to run an entire analysis in a single Python script. Rerunning pipelines are optimized through Nipype caching mechanism and long lasting steps (non-linear warping, perfusion fitting) are executed in parallel. We believe that reproducibility in the neuroimaging field is not possible without making the acquired images and the preprocessing code available to the community. For this reason, Sammba-MRI promotes the sharing of MRI data by providing utility functions to download public small animal brain MRI datasets and relies on it for demonstrating the package capabilities. In order to encourage external contributions, our library source code is hosted on the open collaborative GitHub platform and distributed under the CeCILL v2.1 license, a FOSS license adapted to both international and French legal matters allowing anyone to make changes and redistribute it. Sammba-MRI supports GNU/Linux and Mac OS X operating systems (OS), used by over 70% of neuroimagers (Hanke and Halchenko, 2011). So far, Sammba-MRI is designed for advanced users but documentation is provided to help novices.

## 6. Conclusion

By efficiently combining different existing human and animal neuroimaging tools, Sammba-MRI allows to tackle common processing issues in a fully automated fashion. High quality spatial registration can be easily performed, including template matching, between modalities registration as well as the creation of cohort-specific templates. Sammba-MRI also implements functional and perfusion MRI preprocessing methods and cerebral blood flow estimation for FAIR perfusion images. Emphasis is put on code readability and ease of use to favor contributions from the community.

## Data Availability Statement

The mouse lemur dataset can be automatically loaded through Sammba-MRI or directly from https://nitrc.org/projects/mouselemuratlas for the template and https://openneuro.org/datasets/ds001945 for the original anatomical images. The perfusion dataset will be made publicly available following publication.

## Ethics Statement

The animal study was reviewed and approved by local ethics committees CEtEA-CEA DSV IdF.

## Author Contributions

SB designed the Sammba-architecture and its implementation on Github. SB, NN, and MC contributed code to the project. NN, CG, and MD contributed to data acquisition. SB wrote the first version of the manuscript with input from CG and NN. MC and MD wrote the final version of the manuscript.

## Conflict of Interest

The authors declare that the research was conducted in the absence of any commercial or financial relationships that could be construed as a potential conflict of interest.

## Footnotes

^1^https://www.fil.ion.ucl.ac.uk/spm/software/

^2^http://freesurfer.net/

^3^https://afni.nimh.nih.gov/

^4^https://nipy.org/nibabel/

^5^https://github.com/sammba-mri/sammba-mri

^6^http://gael-varoquaux.info/programming/software-design-for-maintainability.html

^7^https://github.com/lamyj/dicomifier

^8^https://github.com/neurolabusc/Bru2Nii

^9^https://github.com/CristinaChavarrias/Bruker2nifti

^10^https://github.com/SebastianoF/bruker2nifti

^11^https://sammba-mri.github.io/introduction.html#installation

^12^www.fil.ion.ucl.ac.uk/spm

^13^http://spmmouse.org
